# A structural approach to detecting opinion leaders in Twitter by random matrix theory

**DOI:** 10.1038/s41598-023-48682-5

**Published:** 2023-12-08

**Authors:** Saeedeh Mohammadi, Parham Moradi, Andrey Trufanov, G. Reza Jafari

**Affiliations:** 1 Physics Department, Shahid Beheshti University, Tehran, 1983969411 Iran; 2 Center for complex and social data science, Shahid Beheshti University, Tehran, 1983969411 Iran; 3 Institute of Information Technology and Data Science, Irkutsk National Research Technical University, Irkutsk, Russia

**Keywords:** Statistical physics, thermodynamics and nonlinear dynamics, Complex networks

## Abstract

This paper presents a novel approach leveraging Random Matrix Theory (RMT) to identify influential users and uncover the underlying dynamics within social media discourse networks. Focusing on the retweet network associated with the 2021 Iranian presidential election, our study reveals intriguing findings. RMT analysis unveils that power dynamics within both poles of the network do not conform to a “one-to-many” pattern, highlighting a select group of users wielding significant influence within their clusters and across the entire network. By harnessing Random Matrix Theory (RMT) and complementary methodologies, we gain a profound understanding of the network’s structure and, in turn, unveil the intricate dynamics of the discussion extending beyond mere structural analysis. In sum, our findings underscore the potential of RMT as a tool to gain deeper insights into network dynamics, particularly within popular discussions. This approach holds promise for investigating opinion leaders in diverse political and non-political dialogues.

## Introduction

The emergence of Web 2.0 has made a significant shift in the way societies function and how people interact with each other. The wealth of data available on social media platforms has enabled researchers to delve into user behavior and, on a broader scale, examine communities in intricate detail. Countless studies have investigated the formation of echo chambers^1^ within these platforms and their role in exacerbating political polarization across various societies^2^. Understanding these dynamics is imperative due to their undeniable impact on societies. Social media platforms have, in various societies, been exploited for election manipulation. Numerous observational studies have underscored the prevalence of online manipulation during pivotal socio-political events, such as the Brexit referendum^3^, the 2016 U.S. presidential election^4^, and the 2018 Brazilian presidential election^5^.

A correlation between news consumption and voting trends has existed for a long time, predating the era of social media^6–8^. Traditionally, an opinion leader is a person with significant political participation^9^, is exceptionally high in trustworthiness^10^, and spreads a lot of information to their contacts^11^. However, new studies reveal that an opinion leader in a modern era does not need to acquire any of their traditional counterpart’s characteristics^12–14^, and the motive behind their social activism can be rooted in self-presentational goals^15^. It is believed that opinion leaders engage in news consumption and subsequently share it within their social networks^16,17^. Despite the affordability of social media has spurred an increase in political discourse and news sharing^15^, research suggests that it has not fundamentally altered the fundamental structure of how opinion leaders operate. Instead, it has transformed the medium through which these individuals express their opinions and has reshaped the characteristics typically associated with opinion leaders^18,19^. This transformation has created an opportunity for inauthentic users to assume leadership roles within the conversation.

To analyze the information flow within a social media platform such as X (Twitter), it is essential to investigate the retweet network. This network provides a representation of how information is disseminated and propagated across the platform. Numerous researchers have delved into the dynamics of information flow on Twitter through the examination of retweet networks^20–22^. A retweet network is formed by creating directed edges that link the user who authored the original content to those who have shared it through retweets. Consequently, the most influential figure within a retweet network is often considered an opinion leader within that network. In this article, our objective is to pinpoint these opinion leaders by identifying the most prominent nodes within a retweet network related to the 2021 Iranian presidential election.

We introduce a novel approach for identifying these users using Random Matrix Theory (RMT), a widely employed technique in statistical physics for analyzing multivariate data^23,24^. We chose RMT for this study because it offers a deep dive into the network’s structure, extracting meaningful insights from it. Notably, while RMT has been previously utilized to investigate collective behavior in networks such as the stock market network^25–28^ and the global banking network^29^, it has not yet been applied to a social network context. RMT will help us answer vital questions about the prominent users in the retweet network. After identifying such nodes, we move on to analyzing the characteristics of these users including their type (Automated account or genuine users)^17^, their number of followers and followers, and their political stance.

Farsi Twitter is employed as a case study to implement our proposed approach. Specifically, we gather and analyze the retweet network related to trending hashtags concerning the 2021 presidential election. To provide context for readers unfamiliar with Iranian politics, it is crucial to note that the discussion revolves around two opposing viewpoints: users who support a specific candidate or encourage others to vote (referred to as the “Pro community”), and users who are critical of the Iranian government and advocate boycotting the election (referred to as the “Anti community”). This network exhibits high polarization, with a notable presence of bot activity detected within each community^30^. The Persian Twittersphere has been a focal point of numerous studies aiming to decipher the intricacies of interactions among Iranians on this platform and to comprehend the role Twitter plays in Iranian politics^31^. Kermani et al.^32^ highlight the role of Persian Twitter during the 2017 presidential election as a forum for discussing diverse public topics, encompassing both liberal and conservative perspectives. Dehghan et al.^33^ delves into the presence of bots, coordinated influence campaigns, and political astroturfing within Twitter discussions throughout the 2019–2020 Iran protests. Meanwhile, Rahimi et al.^34^ explores how social media platforms, including Twitter, serve as arenas for political confrontation and resistance against state power in post-election Iran. These studies and others^17,30,35^ point to the fact that X (Twitter) plays a significant role as an active Iranian political cyberspace. In this study, we delve into the Persian Twittersphere during a consequential socio-political event, namely the presidential election, with the aim of identifying and analyzing the structure and attributes of opinion leaders. We maintain that this approach can be readily adapted for the examination of other retweet networks. Our methodology leverages RMT for the identification of key figures driving the discourse. Our analysis delves into the roles these users fulfill within their respective communities, scrutinizing their influence at both the global network level and within their interactions with one another. We assert that comprehending this mechanism is pivotal for understanding how narratives take shape during these crucial events.

The structure of this study can be summarized as follows: We initiated data collection by focusing on popular hashtags related to the 2021 Iranian presidential election, spanning from one month before the election to one week after it. Subsequently, we constructed a retweet network using this dataset and employed clustering techniques, along with manual labeling, to identify the primary communities within the network. We then harnessed RMT to pinpoint the most influential users participating in this discussion, with the goal of gaining deeper insights into the discussion’s dynamics as manifested through the network structure.

One key observation arising from our results is that only a small fraction of nodes within the network are responsible for the majority of activities. Furthermore, all the opinion leaders we identified in this discourse have high bot scores^36^, and they belong to specific clusters within each community. These findings collectively suggest the presence of online manipulation within this context. These results indicate that our approach can offer a valuable means to gain a deeper understanding of social networks by solely relying on the network structure, surpassing its structural characteristics, and into the power dynamics within the nodes.

## Methods

### Data collection

Our data collection process is as follows; we manually selected trending hashtags based on activity monitoring tools such as the Twitter app, and Trendsmap. We identified trending hashtags that were related to the election and stored all the tweets and retweets containing those hashtags. Over the course of data collection (April 29, 2021, to June 24, 2021) 97 popular hashtags linked to the Iranian presidential election were collected. More details on the hashtags are available in this reference^30^.

We stored the relevant information in an SQLite database, which selected suitable fields by deconstructing JSON delivered by the API, alongside additional processed data. Python-twitter, a Python wrapper around the Twitter API, is used to call the Standard Search API. We collected tweets that contained hashtags and continuously updated the list of hashtags. All the above progress is managed by our ’Twitter Machine’. This machine handles data collection with separate tasks and prevents duplicated rows. Subsequently, PostgreSQL is used to store the relational data that is gathered by Twitter Machine.

The hashtags related to the election were included in 8818675 tweets by 153115 users that the Twitter Machine gathered. This data was divided into eight different eras in order to examine how user behavior changed over time. We utilized Botometer to gather data on users’ Botscore, Full Automation Probability (Cap), and Fake Followers in order to determine user authenticity^36^. We annotated over 1000 accounts using crowd-sourcing to increase the analysis’s accuracy and derive a threshold to distinguish automated and genuine users in Faris Twitter^17^.

### Retweet Networks

The collected data is turned into directed, binary networks. Each node represents a user that has tweeted a trending hashtag and an edge between users represents the action of retweeting. An edge in the retweet network connects user 2 and user 1 if user 1 ever retweets a post from user 2. To better understand the dynamics of change within the network, the data is divided into different periods, and the network of each time span is analyzed. In our analysis, we distinguish these networks based on their temporal proximity to the day of the election. For example, we refer to the network spanning from 4 weeks before the election to 3 weeks before the election as “4WBE.” In this binary network, if user 1 has retweeted user 2 at any point during this week, we establish a directed edge from user 2 to user 1. It’s important to note that the network’s binary nature means that the frequency of mutual retweets between two users does not impact our analysis.

The community detection in these networks was accomplished using a greedy modularity algorithm. This method relies on optimizing the modularity of clusters and offers improved computational efficiency for large networks when compared to alternative approaches^37^. Afterward, each community is manually investigated and given the appropriate label. More explanation of this procedure is mentioned here^30^.

### Random matrix theory

In the context of random matrix theory analysis, we examine the properties and interactions of various nodes (which represent users). Each node is associated with two key components: an eigenvalue denoted as $\lambda (i)$, and a vector denoted as $|\psi (i)\rangle$, where *N* signifies the number of users or nodes in the retweet network. Within this vector, $\alpha _i^{j}$ represents the jth element associated with the ith nodes’s characteristics. The process involves diagonalizing the matrix that encapsulates the relationships and interactions within the retweet network. Two important metrics are introduced: the Inverse Participation Ratio (IPR) and the Node Participation Ratio (NPR). These metrics help quantify the concentration and participation of nodes within the network’s characteristics.

1. *Inverse Participation Ratio (IPR)* For each node indexed as ’i’, IPR is computed by summing the fourth power of the absolute values of its vector elements. Essentially, it measures how focused or dispersed a node’s characteristics are within its vector.
  1 $$\begin{aligned} IPR(i)=\Sigma _{j=1}^{N} |\alpha _i^{j}|^{4}, \end{aligned}$$
2. *Node Participation Ratio (NPR)*NPR is a similar measure but applied to the elements of the vector representing specific characteristics. For each element ’j’, NPR aggregates the fourth power of the absolute values of that element across all agents.
  2 $$\begin{aligned} NPR_j=\Sigma _{i=1}^{N} |\alpha _i^{j}|^{4}. \end{aligned}$$

IPR informs us about the degree of concentration of a specific characteristic within an eigenvector. A high IPR suggests that the characteristics are heavily focused on a few elements of the vector. On the other hand, NPR reveals how actively users participate in the network’s trend for a specific characteristic. High NPR values for certain elements of the vector indicate that many users are actively involved in that particular aspect of the network’s behavior.

Furthermore, The IPR calculated for the eigenvector with the highest eigenvalue sheds light on the overall trend or behavior of the entire network. It identifies the node with the most concentrated characteristics. High NPRs associated with large eigenvalues indicate that those users are key participants in driving the network’s behavior concerning the specific characteristic under examination. In summary, this analytical approach provides insights into how various characteristics or properties are distributed among agents within a retweet network. It enables us to pinpoint the concentration of these properties within individual nodes and identify the influential participants shaping the network’s behavior.

## Results and discussion

**Figure 1:**
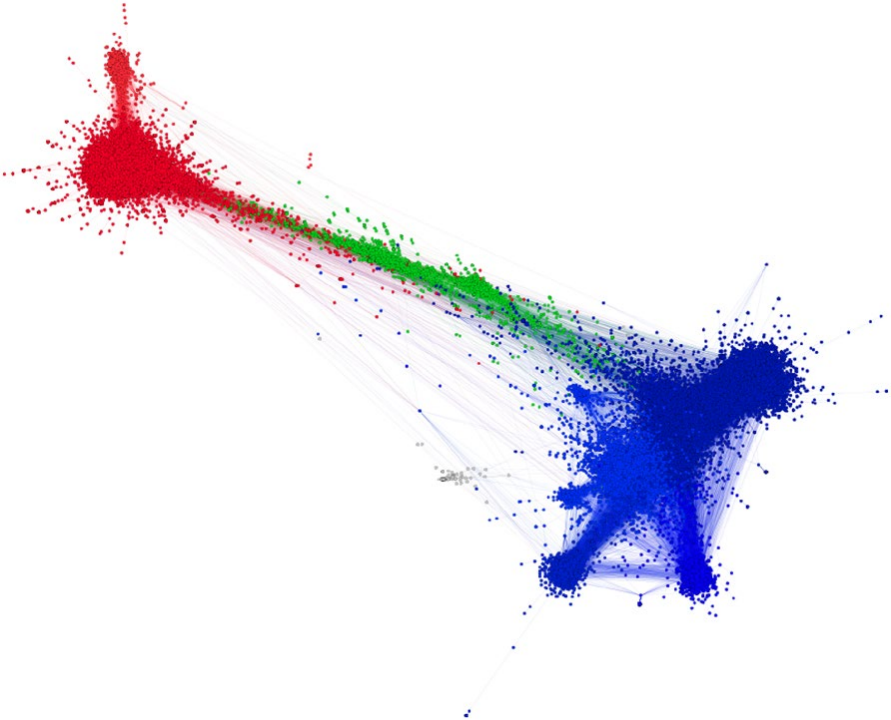
The visualization of the retweet network from 4WBE. The node’s colors represent its community. The two main poles are the Anti (blue) and Pro (Red) communities. The visualisation was done with Gephi 0.9.7 202208031831.

We begin our analysis by detecting three major communities in each network. It was previously detected that these networks are polarized (^30^). Hence, the major communities in the networks are the two poles and the community between them. The community at one end of the graph, among all networks, consists of users who were against voting in this election, and the nodes at the other end are the ones who declared they would participate in the election, therefore, in this research, they are regarded as Anti and Pro communities respectively. The middle community works as a gray zone in this research. It is important to note that from previous research, it is gathered that the users in the middle community have different views about the election from the two poles. An illustration of one of the networks is shown in Fig. 1. The color and size of each node represent its community and in-degree respectively. The color red represents the Pro community, and the color blue represents the Anti community. The layout of the graph is based on the forceAtlas2 algorithm^38^.

**Figure 2:**
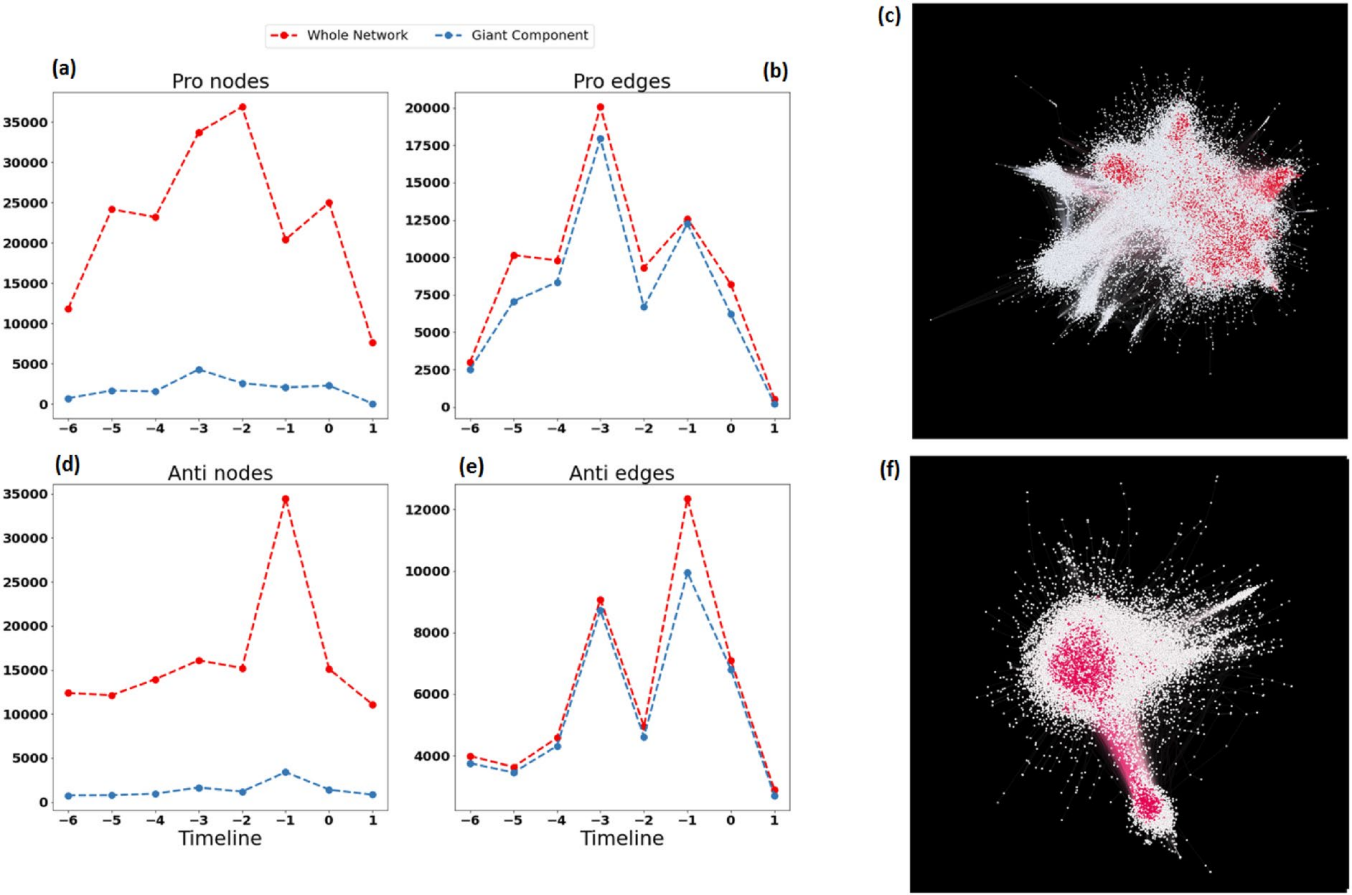
(**a**) The number of nodes for the Pro community (blue) and the giant component (orange) over time. (**b**) The number of edges for the Pro community (blue) and the giant component (orange) over time. (**c**) An illustration of the accumulated Pro communities. The dark nodes were in the giant component with a low degree. (**d**) The number of nodes for the Anti community (blue) and the giant component (orange) over time. (**e**) The number of edges for the Anti community (blue) and the giant component (orange) over time. (**f**) An illustration of the accumulated Anti communities. The network visualizations were done with Gephi 0.9.7 202208031831.

Further investigations on these communities revealed that there are a small number of nodes that are all connected in each pole (Anti and Pro community), that are responsible for the majority of the edges (retweets). In other words, the giant component of each community consists of less than 10% of the total number of nodes in the networks and more than 90% of the edges. Figure 2(a) and (d) show the number of nodes in the network (blue line) and the number of nodes in the giant component (red line) in the Pro and Anti communities respectively. Figure 2(b) and (e) depict the number of edges for the whole network (blue line) and the giant component (red line) in the Pro and Anti community. These statistics are demonstrated over the timeline of data collection for each network. It is evident that while the giant component consists of only a fraction of the nodes, is responsible for almost all of the edges in the network. This trend is consistent throughout the timeline. Figure 2(c) and (f) are an illustration of the accumulated networks for the Pro and Anti community over the timeline. The red nodes are in the giant components of the communities. This structure resembles an atom, with giant components imitating a nucleus, dense, focused, and a small number of particles interacting with each other, and the rest of the nodes appear as electrons, very sparse and distanced from the core. Therefore, from now on the giant component in each network is referred to as “the core” and the rest of the nodes are referred to as “the cloud”. The notion that the majority of activity in these communities is done by a small group of nodes, points to the presence of an echo chamber. An echo chamber refers to a setting in which an individual is surrounded by like-minded individuals who reinforce and amplify their beliefs, often fostering radicalization and intensifying those convictions^39^. The core is in control of the structure of the communities even though it has far fewer “players” than the rest of the network. In an attempt to find the leaders of the discussion, one should search the core of each community.

**Figure 3:**
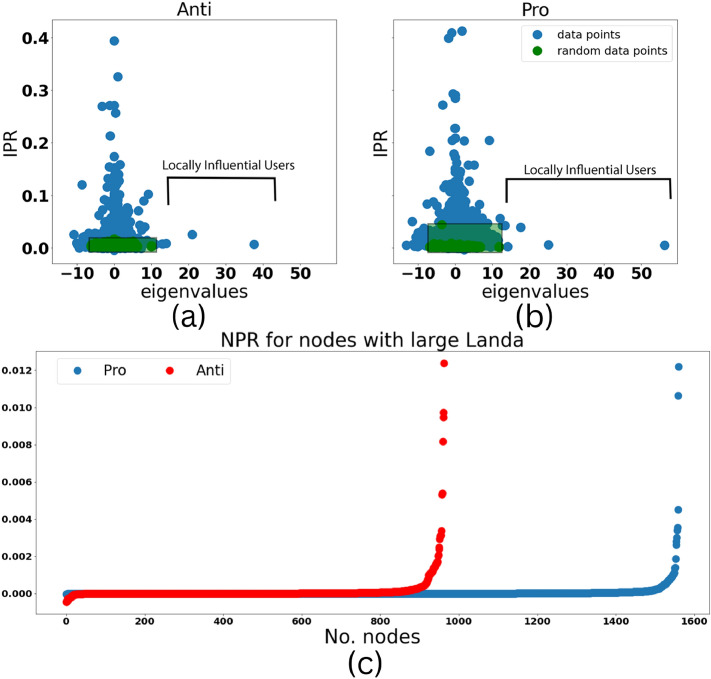
IPR to the eigenvalue of the Anti (**a**) and Pro (**b**) community in the network of 3 to 4 weeks. The red dots belong to the shuffled network. (**c**) NPR for the nodes with eigenvalues higher than random. Red dots belong to the Anti community and blue dots belong to the Pro community.

Random Matrix theory is used to detect the more participated and influential nodes in each graph. First, the eigenvalues of the adjacency matrix and IPR of each eigenvector are calculated. Fig.3(a) and (b) show the IPR of each eigenvalue for the Anti and Pro community respectively in 4WBE. The same analysis is done for a random graph with the same number of nodes and edges to compare the results and discover the abnormal behaviors. These plots show two important findings. First, the points with high IPR and eigenvalues close to zero are evidence of very high modularity in the graph. Second, the points with high eigenvalues and low IPR are evident of locally influential users. In other words, there is no particular leader within these graphs but a handful of users that are influential within each cluster of the graph. Fig.3(c) shows the NPR of the nodes with eigenvalues higher than the maximum eigenvalue for the random graph. A few nodes here are exhibiting significantly larger NPR than other nodes which indicates that these users are dominant among the whole graph as well. It is important to note that other graphs in different snapshots show the same behavior during this investigation.

Further examination into the nodes, which from now on we would refer to as “leaders”, show that these are not what a social media analyst would call “an influencer” since their follower count does not pass a couple thousand. They are not necessarily the most retweeted accounts. On the other hand, according to RMT analysis, they are vital actors in the graph. Table 1 shows the result of the manual examination of the leaders in 4WBE. The usernames are removed for the sake of privacy and according to Twitter developers’ terms of conduct. In the column “Type”, a label based on the recent activity of the users is given to them. An activist is a user who only has politically related posts in their last 100 tweets. An individual would be someone who uses Twitter for personal use as well as a platform to discuss their political ideology. Most of the users in the leader’s group are devoted activists who only use Twitter to propagate their beliefs. This behavior is consistent in all networks.

**Table 1:** The results of the manual examination of the leaders in Pro and Anti Community in 4 weeks before the election.

Pro				Anti			
id	Follower count	Type	Cap score	id	Follower count	Type	Cap score
1	2k	Activist	0.81	2	2k	Activist	0.80
3	843	Activist	0.73	4	4k	Individual	0.38
5	142	Activist	0.74	6	1k	Activist	0.81
7	3k	Activist	0.81	8		Deleted	
9	2k	Individual	0.81	10	497	Activist	0.68

**Figure 4:**
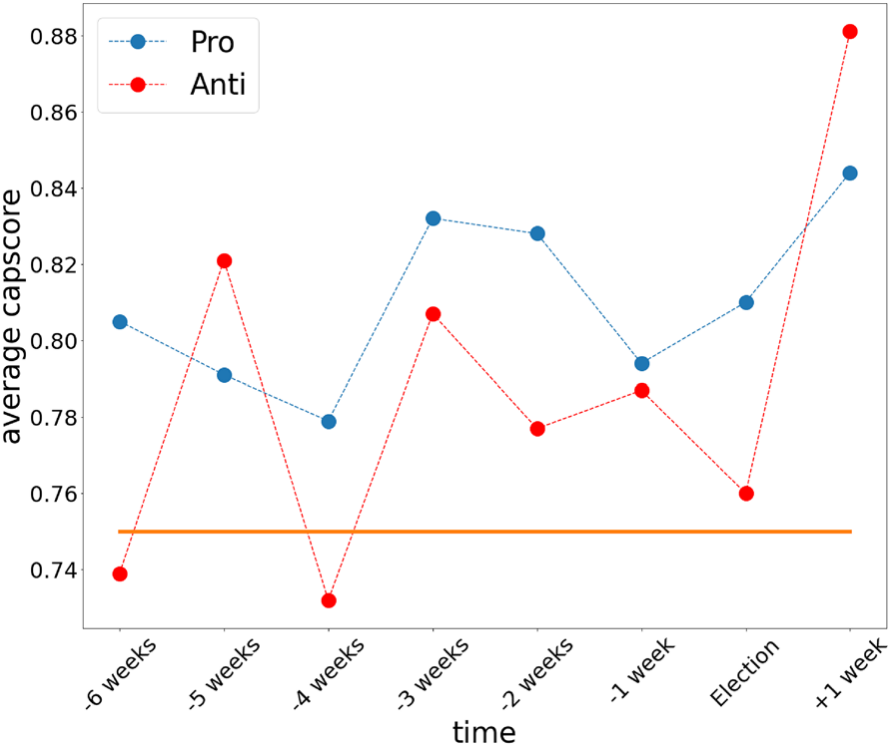
The average cap score of leaders during the data collection. The red line is the average cap score of the Anti community and the blue line is for the Pro community.

Another important finding is the significantly high cap score in leaders. Fig. 4 illustrates the average cap score in each week of the data collection, showing that in each week the leaders of both communities are well above the threshold (orange line) for bots. It is evident that since the users in the leader’s group change over time in both communities, the cap score changes as well. One interesting fact is that in both communities the cap score is significantly higher in 1WAE, which points to the fact that genuine users have lost interest in the topic after the election.

**Figure 5:**
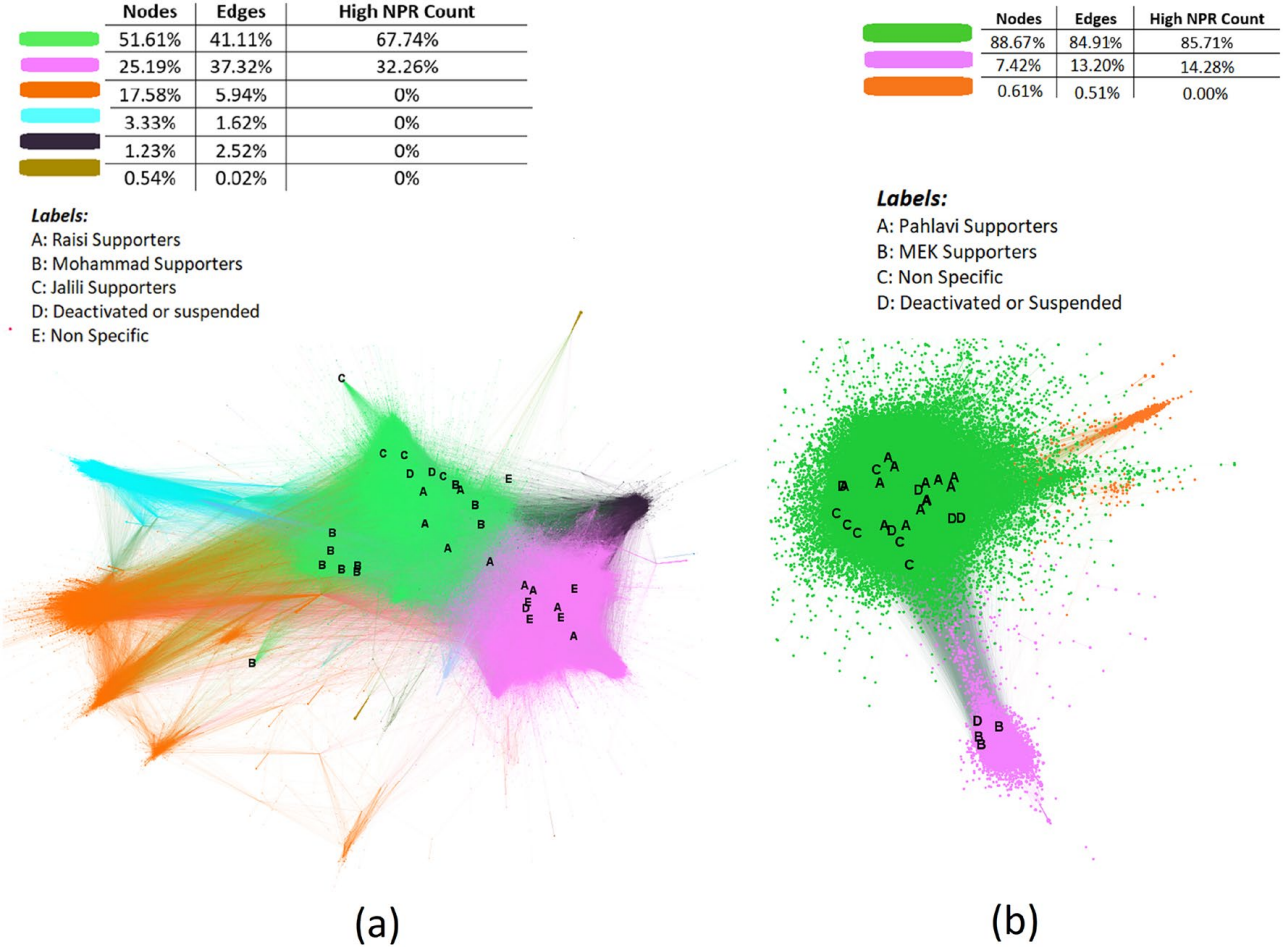
(**a**) The accumulated Pro community. influential nodes are labeled. (**b**) The accumulated Anti community. influential nodes are labeled. Each node’s color indicates its community and size by its in-degree. The visualisation was done with Gephi 0.9.7 202208031831.

The leaders are investigated in the whole graph, to have a better grasp of their nature. Fig. 5 shows the whole graphs of Anti and Pro with the leaders of each group labeled by their political views. nodes with the same color belong to the same sub-community. It is illustrated that the leaders are only active in the two biggest sub-clusters in each graph and the ones with the same political ideology are closer together. These findings align with what was discovered in the RMT analysis that these graphs are highly modular and there are few nodes in each cluster with the same agenda that is particularly more influential than other nodes.

**Figure 6:**
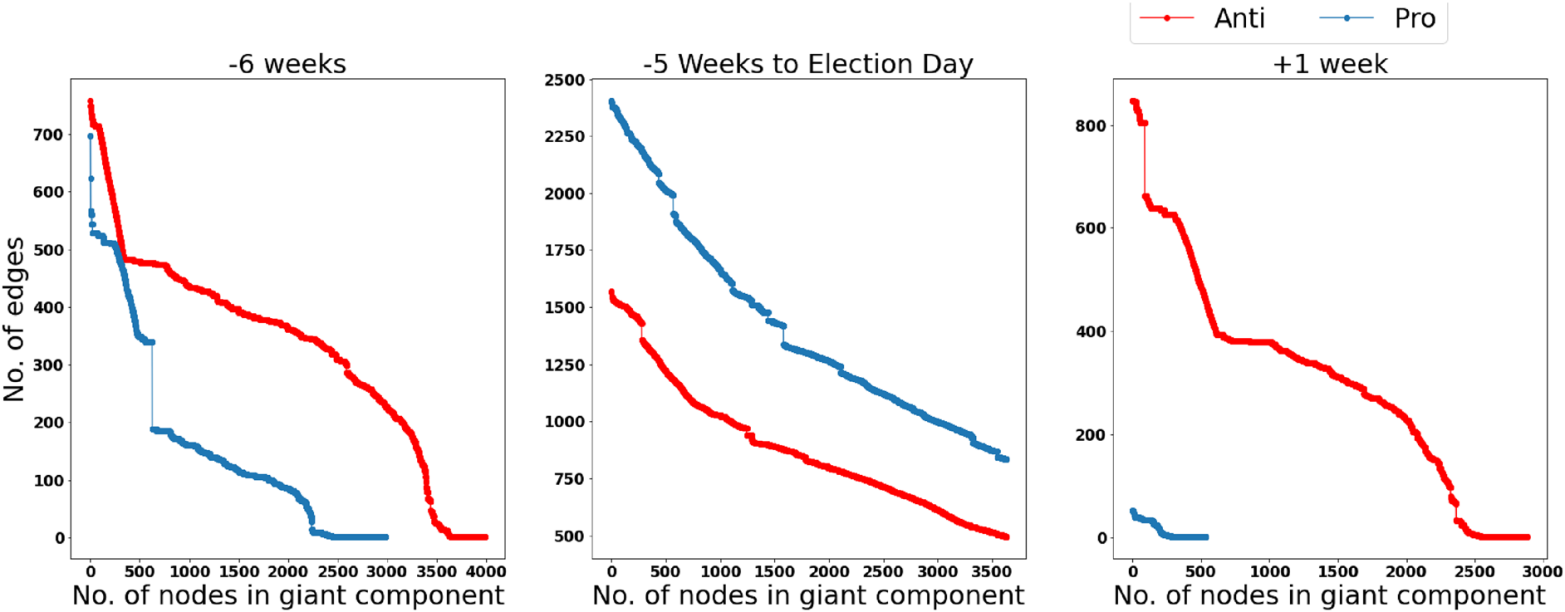
The number of nodes in the core of Anti (blue) and Pro (red) communities as edges are removed based on their betweenness centrality in descending order.

To calculate the strength of each core during the timeline, the edges of each community are attacked based on their’s betweenness centrality. The algorithm is as follows; first, the betweenness centrality of each edge in the core is calculated; then the edge with the highest betweenness centrality is removed and the number of nodes in the giant component is stored. This process continues until there is no edge left in the graph. It is clear, that with each attack the size of the giant component is reduced, however, there is a point at which this reduction is sharper and more rapid than the rest. If this sharp reduction happens later in the attack, the graph is believed to be more resilient and more structurally strong. This analysis was done on Pro and Anti communities in each snapshot. Results show that the Anti community showed more strength in 5WBE and 1WAE, whereas the Pro community was stronger in the times in between. Fig. 6 depicts the number of nodes in the giant component in the Pro (red) and Anti (blue) communities after each attack. The plot for 5WBE (a) and 1WAE (c) is shown alongside the plot of an average of the weeks in between (b), which all had shown similar behavior.

The same analysis based on random matrix theory is done to the whole network and discovered that in the initial week of data gathering most of the Anti’s influential nodes were also the networks, however, after that, the role is reversed and most of the Pro influential nodes are listed in the whole networks’ influential nodes. This behavior is consistent until the week after the election when the Anti community’s leaders are once again among the network leaders. The sustained prevalence of the Anti group on Farsi Twitter, consisting primarily of anti-government activists, is noteworthy. Their consistent presence suggests a longstanding influence within the platform. In contrast, the Pro community exhibits a different pattern, with a sudden surge in activity. However, this surge appears to be situational, occurring primarily in specific trends and discussions that hold particular political significance for them, such as the presidential election. This nuanced dynamic implies that the Anti community maintains a more continuous and pervasive engagement on the platform, while the Pro community strategically seizes control in contexts that are politically significant. It’s important to note that these dynamics may vary in other contexts or events outside the analyzed event.

In summary, the RMT analysis uncovers interesting power dynamics. In both clusters, a few users seem to function as leaders are are highly influential. The NPR analysis further reveals that these influential users not only possess a high eigenvalue within their respective clusters but also wield substantial influence across the entire network. This trend appears consistent throughout the observation period. Although the specific users may change from week to week, the average cap score for this influential group consistently remains above the threshold in most weeks. Furthermore, our systematic edge attack analysis offers valuable insights. It suggests that the Pro community only exhibits greater resilience as the election date draws nearer, in contrast to the Anti community. This implies that the Anti community maintains a more stable presence within this social space, while the pro-community tends to surge in influence primarily when the conversation about the election is actively taking place.

The presence of online manipulators on Farsi Twitter has been previously documented in existing literature^17,30,32,35^. Our findings underscore the capability of RMT to unveil the underlying power dynamics associated with these manipulative activities. The consistently elevated cap scores assigned to influential users in both major communities reaffirm the existence of automated or semi-automated users in this discourse, mirroring patterns observed in prior political conversations within this medium^17^. However, the RMT analysis introduces nuances to this understanding. The identified leaders exhibit a high eigenvalue, low IRP, and high NPR. This signifies that these nodes are not only influential and central figures in the network but also possess a broad impact on various characteristics. In contrast to scenarios with low IRP alone, the high NPR indicates that these nodes actively contribute to and shape specific aspects of the network’s behavior. They transcend the role of general influencers and specialize in certain features or trends within the network. In the context of a retweet network, these nodes may represent users with widespread influence on diverse topics. Yet, they are also recognized for actively engaging in and steering specific trends or discussions. This dual role allows them to contribute to the overall dynamics of the network while exerting a targeted influence on particular facets of the shared or discussed content. It’s noteworthy that these users, identified solely through RMT analysis, would not be considered influencers in the common meaning of the word, as their follower count may not be particularly high. Their influence is discerned through their role in shaping the network’s behavior, emphasizing the importance of alternative metrics in understanding online influence dynamics.

## Conclusion

In conclusion, this paper has introduced Random Matrix Theory (RMT) as a novel approach for identifying influential users and uncovering the underlying dynamics of discussion networks within social media. Our study focused on the retweet network related to the 2021 Iranian presidential election, leading to key observations, such as the power dynamics within both poles of the network deviate from a “one-to-many” pattern. Instead, a select few users exert significant influence within their clusters and wield substantial sway over the entire network. Notably, these users wouldn’t be identified through conventional methods for spotting influencers, as they don’t exhibit traits associated with “traditional influencers.” However, RMT analysis reveals their prominent impact on the network’s behavior.

While other research has emphasized approaches such as text analysis and network analysis^40–42^ to analyze user behavior in discussion networks, our study distinguishes itself by employing RMT to gain a deeper understanding of information propagation within this medium. This innovative method sheds light on the dynamics at play and, enhances our grasp of network dynamics in social media discussions.

The findings presented in this study highlight the potential of Random Matrix Theory (RMT) as a powerful tool for gaining nuanced insights into network dynamics by focusing solely on the examination of the network’s structure. Looking forward, there are several promising avenues for future research and development using this approach.

Firstly, the application of RMT can be extended to investigate the attributes of opinion leaders in various political and non-political dialogues. By identifying and understanding the influence dynamics of opinion leaders, we can enhance our comprehension of information dissemination processes and influential figures across diverse topics and discussions within social media. Secondly, the exploration of RMT on friendship networks associated with similar conversations could provide valuable comparative insights. Contrasting structural leaders identified by RMT with opinion leaders within friendship networks may unveil distinctions in how influence operates within different social contexts. This comparative analysis could contribute to a more comprehensive understanding of social influence dynamics. Additionally, future research could benefit from the adoption of a more sophisticated clustering approach to improve the identification of distinct communities within the network. Fine-tuning the clustering methodology would enhance our ability to discern and characterize the various subgroups present in the social media discourse.

However, it’s essential to acknowledge a limitation of this study, specifically its relevance primarily to popular discussions. The effectiveness of the RMT approach depends on the condition that the network has a giant component. This may render the method less applicable when local user interactions do not translate into significant global influence. To address this limitation, we have excluded small clusters from our analysis. Future work could explore ways to adapt the approach for diverse discussion contexts, ensuring its applicability across a broader range of social media interactions. Additionally, efforts could be directed toward refining the exclusion criteria for small clusters to strike a balance between computational efficiency and capturing meaningful network dynamics.

## Data Availability

The datasets generated and analyzed during the current study are available in the following repository, https://ccnsd.ir/research_projects/twitter/.
